# Genome sequences of nine *Clostridium scindens* strains isolated from human feces

**DOI:** 10.1128/mra.00848-24

**Published:** 2024-11-04

**Authors:** Francelys V. Fernandez-Materan, Kelly Yovani Olivos-Caicedo, Steven L. Daniel, Kimberly K. O. Walden, Christopher J. Fields, Alvaro G. Hernandez, Joao M. P. Alves, Jason M. Ridlon

**Affiliations:** 1 Carl R. Woese Institute for Genomic Biology, University of Illinois Urbana-Champaign, Urbana, Illinois, USA; 2 Department of Animal Sciences, University of Illinois Urbana-Champaign, Urbana, Illinois, USA; 3 Department of Parasitology, Institute of Biomedical Sciences, University of São Paulo, São Paulo, Brazil; 4 Department of Biological Sciences, Eastern Illinois University, Charleston, Illinois, USA; 5 Roy J. Carver Biotechnology Center, University of Illinois Urbana-Champaign, Urbana, Illinois, USA; 6 Division of Nutritional Sciences, University of Illinois Urbana-Champaign, Urbana, Illinois, USA; 7 Cancer Center at Illinois, Urbana, Illinois, USA; 8 Center for Advanced Study, University of Illinois Urbana-Champaign, Urbana, Illinois, USA; 9 Department of Microbiology and Immunology, Virginia Commonwealth University, Richmond, Virginia, USA; Wellesley College Department of Biological Sciences, Wellesley, Massachusetts, USA

**Keywords:** *Clostridium scindens*

## Abstract

*Clostridium scindens* is an important member of the gut microbiome. Strains of *C. scindens* are model organisms for bile acid and steroid metabolism studies. The genome sequences for nine *C. scindens* strains isolated from human feces are reported. Genomes ranged from 3,403,497 to 4,318,168 bp, 46.5% to 48% G+C content, and 3,386 to 4,137 protein-coding total genes.

## ANNOUNCEMENT

*Clostridium scindens* ATCC 35704 and VPI 12708 have served as model strains for the study of bile acid 7α-dehydroxylation (1–6). Additional 7α-dehydroxylating strains were isolated in the 1990s, later classified as *C. scindens* (1–3, 7–9), but publicly accessible genomes for these strains are lacking.

*C. scindens* strains were grown in anaerobic Trypticase Soy Broth (TSB) at 37°C. Once culture purity was established by plating on anaerobic Trypticase Soy Agar (TSA) with 10% sheep’s blood, strains were verified as *C. scindens* based on their reported DNA sequences. Genomic DNA (gDNA) was obtained as described (10), but without bead beating. Briefly, TSB-grown cells were pelleted, washed, resuspended in TE buffer (10 mM Tris, 1 mM ethylenediaminetetraacetate, pH 7.6), and incubated (37°C, 1 h) with lysozyme (2 mg/mL final concentration). Next, proteinase K (1 mg/mL final concentration) was added and incubated (37°C, 30 min), followed by sodium dodecyl sulfate (1% final concentration) addition and incubation (60°C, 20 min). Buffered phenol was added to digested cells (1:1, vol/vol) and vortexed. The aqueous phase was cleaned with phenol/chloroform/isoamyl alcohol (25:24:1, vol/vol/vol). DNA was precipitated with 1/10 vol 3 M sodium acetate and 2 vol cold absolute ethanol, pelleted by centrifugation, and washed with 70% ethanol. DNA pellets were dried by vacuum centrifugation, dissolved in TE buffer, and quantified by Nanodrop (Thermo Scientific, Delaware, USA).

Strains I10-S077 were sequenced using prepared gDNA with both Oxford Nanopore Technology (ONT) long reads and Illumina short reads. ONT libraries were produced using unsheared gDNA, NBD114 barcoding, and 1D Library SQK-LSK109 kits, then sequenced on a SpotON-R9.4.1 FLO-MIN106 flowcell using a GridIONx5 sequencer for 48 h. Real-time base-calling and demultiplexing were performed with Guppy 4.0.16 using the high-accuracy mode. Illumina libraries were constructed with the Roche Kapa HyperPrep Kit and sequenced on a MiSeq flowcell (Illumina, California, USA) for 251 cycles from both ends using a MiSeq Nano kit (Illumina, California, USA). The resulting FASTQ files were demultiplexed with Illumina bcl2fastq v2.20 Conversion Software.

JCM strains were sequenced using prepared gDNA with long-read PacBio technology by first shearing gDNA into 13-kb fragments using a Megaruptor 3 (Diagenode, Denville, NJ). Libraries were constructed with the PacBio SMRTBell Express Template Prep Kit 3 and sequenced on a PacBio Sequel IIe (California, USA), using the circular consensus sequencing mode with a 30-h movie time and SMRTLink V11.0 parameters (--ccs --min-passes 3 --min-rq 0.99).

All sequencing reads were evaluated by FastQC v0.11.9 (11) prior to assembly. ONT reads were first adapter-cleaned with Porechop v0.2.3 (12) using “—discard-middle” and then seqtk v1.3 (https://github.com/lh3/seqtk) to remove reads <1 kb. MiSeq reads were trimmed with Trimmomatic v0.38 (13) using default settings. PacBio reads were first filtered to retain reads between 1 and 50 kb with seqkit v0.12.1 (14) and then down-sampled to 50× expected genome coverage using filtlong v0.2.1 (12), using default parameters for both.

Assemblies for strains I10-S077 were generated with Unicycler v0.5.0 (15) in the hybrid mode to use the ONT and Illumina reads, outputting the “moderate” versions (15). They were assessed for completeness with BUSCO v3.1.1 (16) and the Clostridia odb9 data set containing 254 single-copy BUSCOs for comparisons. PacBio-only assemblies of JCM strains were generated with Flye v.2.8.2 (17), using “--hifi-error 0.003.” They were assessed for completeness with BUSCO v.4.1.4 (16) using the *Clostridia* odb10 data set containing 247 single-copy BUSCOs for comparisons. All assemblies were annotated with Prokka v1.14.6 (18). Default parameters were used for all software.

**TABLE 1:** Genome assembly metrics for nine strains of *Clostridium scindens*

*Clostridium scindens* strain	Source^*a*^	Sequencing technology^*b*^	Number of reads	Read *N*_50_	SRR accession number	Assembly method	Genome size (bp)	*N*_50_ assembly value	G+C (%)	BUSCO scores (%)	Completion level^*c*^	Gene (total)	rRNA	tRNA	tmRNA	Genome assembly number
JCM10419 (Y1113)	JCM	PB(SE)	111,907	11,808	SRR27029003	Flye	3,940,699	3,940,699	47.5	99.6	NC	3,682	12	57	1	ASM3353943v1
JCM10420 (M-18)	JCM	PB(SE)	127,859	11,005	SRR27029002	Flye	4,020,045	4,020,045	47.5	99.6	Closed	3,768	11	57	1	ASM3353941v1
JCM10422 (TH-82)	JCM	PB(SE)	95,020	10,513	SRR27029001	Flye	3,501,106^*d*^	3,222,502	46.6	100	Contigs (2)	3,390	12	56	1	ASM4093202v1
JCM10423 (36-S)	JCM	PB(SE)	210,152	9,420	SRR27029000	Flye	4,306,053^*e*^	4,306,053	47.0	100	NC	4,057	11	57	1	ASM3353951v1
I10	VCU	ONT(SE) Illum(PE)	496,293 2,828,088	5,623 249	SRR28053600 SRR27028999	Unicycler	3,435,295	3,435,295	46.5	100	Closed	3,602	12	56	2	ASM3353949v1
MO32	VCU	ONT(SE) Illum(PE)	197,510 2,573,362	12,723 250	SRR28053599 SRR27028998	Unicycler	3,929,075	3,929,075	48.0	99.6	Closed	3,691	11	57	1	ASM3353945v1
NTI82	VCU	ONT(SE) Illum(PE)	413,294 2,250,767	9,173 250	SRR28053598 SRR27028997	Unicycler	4,318,168	4,318,168	47.0	99.6	Closed	4,137	11	59	1	ASM3353953v1
S076	VCU	ONT(SE) Illum(PE)	335,698 3,007,980	9,706 250	SRR28053597 SRR27028996	Unicycler	4,290,604^*f*^	4,290,604	47.5	99.6	Closed	4,103	11	58	1	ASM3353947v1
S077	VCU	ONT(SE) Illum(PE)	233,133 2,174,635	8,120 250	SRR28053596 SRR27028995	Unicycler	3,403,497	1,800,687	46.5	100	Contigs (5)	3,386	12	57	2	ASM4093201v1

^*a*^ Strains were obtained as actively growing plate cultures from the Japanese Culture Collection (JCM) or as frozen 30% glycerol stocks from Virginia Commonwealth University (VCU) and, once received, were transferred to anaerobic TSB for routine cultivation at 37°C. All strains were originally isolated in the 1990s from fecal samples of healthy adult humans by Fusae Takamine and colleagues at the University of the Ryukyus (see references 1–4 and the JCM website, https://jcm.brc.riken.jp/, for more information about these strains). In the 1990s, strains Y-1113, M-18, TH-82, and 36S (each strain was isolated from a different individual) were deposited at JCM by F. Takamine. Strains I-10, MO32, NT182, S076, and S077 were sent by F. Takamine to Dr. Phillip B. Hylemon at VCU; there is no published information on whether these strains were derived from one individual or multiple individuals.

^*b*^ Sequencing technologies used for each strain were PB (Pacific Biosciences), Illum (Illumina), and ONT (Oxford Nanopore Technologies). Library layout read specified as single-end (SE) or paired-end (PE) sequencing.

^*c*^ Best completion levels provided by assemblers were considered. Contigs, chromosome is split into fragments (parenthetical value indicates the number of chromosomal fragments); NC, chromosome is in one fragment, but not circularized; and Closed, chromosome is in one fragment and circularized.

^*d*^ Sample JCM10422 was assembled from 100× coverage PacBio reads, while all other PacBio reads were down-sampled to 50× genome coverage.

^*e*^ Sample JCM10423 was assembled with 99.9RQ PacBio reads, while all other PacBio reads used were 99.0RQ.

^*f*^ Sample S076 produced several contigs that could be merged with Bandage v0.8.1 (19) using the GFA file to produce a single 4.2 Mb closed chromosome.

## Data Availability

Project data are available under BioProject accession number PRJNA1026650 and were deposited in GenBank with individual genome and BioSample accession numbers (Table 1). Genome annotation files are available in the Illinois Data Bank (https://doi.org/10.13012/B2IDB-1661997_V1).
